# The relationship between perceived sense of control and visceral adipose tissue - the North Texas Healthy Heart Study

**DOI:** 10.1186/1751-0759-5-12

**Published:** 2011-09-13

**Authors:** Roberto Cardarelli, Sandy-Asari Hogan, Kimberly G Fulda, Joan Carroll

**Affiliations:** 1 Department of Behavioral and Community Health, School of Public Health, University of North Texas Health Science Center at Fort Worth, 3500 Camp Bowie Blvd, Fort Worth, TX 76107, USA; 2 Primary Care Research Center/Texas Prevention Institute, University of North Texas Health Science Center at Fort Worth, 3500 Camp Bowie Blvd, Fort Worth, TX 76107, USA; 3 Department of Family Medicine, Texas College of Osteopathic Medicine, University of North Texas Health Science Center at Fort Worth, 3500 Camp Bowie Blvd, Fort Worth, TX 76107, USA; 4 Department of Integrative Physiology, Graduate School of Biomedical Sciences, University of North Texas Health Science Center at Fort Worth, 3500 Camp Bowie Blvd, Fort Worth, TX 76107, USA

**Keywords:** Sense of control, visceral adipose tissue, cardiovascular, psychosocial

## Abstract

**Background:**

The purpose of this study was to assess the relationship between one's sense of control and visceral adipose tissue.

**Methods:**

This cross-sectional study recruited 571 subjects (45 years and older) who were asymptomatic of CHD from Fort Worth, Texas from 2006 to 2008. Subjects completed a questionnaire, body measurements, a multi-slice computed tomography scan to assess for visceral adipose tissue (VAT) centered at the L4L5 spinal interspace, and serum chemistries. The natural log of L4L5 VAT (lnVAT) was used in all analyses to achieve normality of the data with final analyses including 506 participants. Linear regression was used to estimate unadjusted and adjusted beta-coefficients and standard errors for the association between sense of control and lnVAT.

**Results:**

A total of 506 participants were used in the data after adjusting for normality of the data. An increase in sense of control was associated with a decrease in lnVAT in the unadjusted (p < 0.001) and adjusted (p = 0.03) models. Other factors significantly associated with lnVAT in the adjusted model include age, BMI, male gender, non-Hispanic African American, and diet.

**Conclusions:**

Sense of control remained as an independent factor associated with visceral adiposity despite adjusting for traditional cardiovascular risk factors, including BMI. Future studies should focus on establishing a causal relationship between sense of control and visceral adiposity.

## Background

The burden of excessive weight and its association with adverse health outcomes has been extensively examined by researchers over the years. There has been an increasing amount of interest on the accumulation of visceral adipose tissue (VAT), which is a form of body fat that surrounds various organs of the body. This interest has arisen since VAT is more metabolically active compared to other types of adipose tissue and is known to impact many metabolic abnormalities that have been associated with excessive weight gain [1-10]. Moreover, VAT has been associated with hypertension, insulin resistance, and dyslipidemia [11]. Major factors affecting VAT include sex-hormone imbalance, aging, excessive intake of sucrose, weight gain, genetic makeup, and lifestyle factors including the lack of physical activity, alcohol consumption and cigarette smoking [11]. However, little research has been conducted on the independent influence of psychosocial factors, such as sense of control, on VAT.

Control constructs, such as self-efficacy [12], perceived control [13], and helplessness [14], are examples that have sought to explain and elucidate how "control" interacts with human behavior. Interventions that seek to improve health outcomes must consider the barriers and facilitators to behavioral change; one of which is one's sense of control. Sense of control is the belief that one masters, controls, and shapes one's own life. It is related to self-efficacy and locus of control and has been found to have adaptive effects which influences emotional wellbeing, physiological changes of stressors, ability to cope with stress, performance, pain, and ability to making difficult behavior changes [15,16].

According to the *Theory of Planned Behavior *a person's attitude, social norm, and perceived behavioral control influences his or her intentions for behavioral change [17-19]. This implies that individuals are more likely to engage in behaviors over which they have control. Thus, perceived behavioral control is determined by the perceived presence or absence of resources and opportunities and their ability to induce or hinder performance [19]. More research is needed to study the direct and indirect physiological health consequences of sense of control that are mediated through health behaviors. One such objective measure is VAT.

The purpose of the proposed study is to elucidate the association between one's perceived sense of control and VAT, regardless of chronic disease status, smoking behavior, and socioeconomic status.

## Methods

### Study population

**Figure 1:**
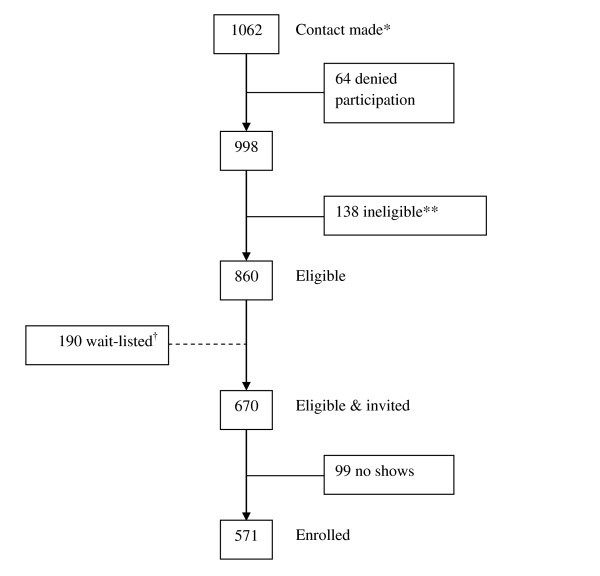
**Recruitment flow diagram**. *Recruited or participants called the research office. **Based on inclusion/exclusion criteria. †Individuals were wait-listed if their respective race/ethnicity blocks were full at the time contact was made.

The North Texas Healthy Heart (NTHH) study is a cross-sectional study involving a convenience sample of 571 non-Hispanic whites, non-Hispanic African Americans, and Hispanics/Latinos recruited from 12 participating sites of the North Texas Primary Care Practice-Based Research Network (NorTex) from April 2006 to May 2008 (USA). The 12 family medicine/internal medicine clinic sites that participated in the NTHH study included 4 academic community-based clinics, 3 community health centers, 4 solo-practitioner private practices, and 1 federally-qualified health center. Potential participants were either actively recruited by research coordinators within clinics, referred to the research office by clinic office staff, providers, or active participants, or potential participants directly contacted research staff through advertisements posted in local community newspapers or flyers. Participants were eligible for the study if they were 45 years of age or older, self-identified as non-Hispanic white, non-Hispanic African American, or Hispanic/Latino, and had no history of self-reported cardiovascular disease (coronary artery disease, peripheral arterial disease, history of myocardial infarction or stroke, or congestive heart failure), renal failure, or liver failure. All participants were screened for eligibility either on-site or via phone from a centralized NorTex research office located within the University of North Texas Health Science Center. Initial contact was made with 1,062 individuals, with 860 meeting eligibility criteria. Of those who were eligible, 670 were consecutively invited to participant based on availability of research slots and 571 agreed to participate and the remaining were wait-listed, representing an 85% recruitment rate (Figure 1). Participants were reimbursed $60 for their time and effort in participating in the study. All study procedures were approved by the University of North Texas Health Science Center and JPS Health Network Institutional Review Boards.

### Study procedures

All consented participants underwent a 1-hour face-to-face interview. Women, except for those with a history of hysterectomy, underwent a urine pregnancy test, as pregnancy is a contraindication to computed tomography scanning. There were no positive urine pregnancy tests among the study participants. Participants then completed weight, height, waist/hip circumferences, and blood pressure measures (millimeters of mercury [mmHg]). Height was measured to the nearest 0.25 inch, and weight was measured to the nearest 0.25 lb using a standard balance scale. Height and weight measurements were used to calculate a body-mass index for each subject using the Quetelet's equation (kg/m^2^) [20]. Automated Welch Allyn^© ^sphygmomanometers were used to measure heart rate and systolic and diastolic blood pressures in each arm using a size-appropriate cuff. The measures were taken after the participant was seated quietly for 5-minutes with both feet flat on the floor and the back comfortably supported. An average heart rate and systolic and diastolic blood pressure was calculated for each subject based on two separate measures.

### Demographic and health behavior measures

The study utilized standardized questions from the Behavioral Risk Factor Surveillance System to collect a selected number of demographic and health behavior information. Age was registered as a continuous variable (years). Race/ethnicity was self-reported and categorized as non-Hispanic white, non-Hispanic African American, Hispanic, and other. Education was measured by the question, "What is the highest grade or year of school that you completed?" Responses were then categorized as "less than high school", "high school graduate/General Equivalency Diploma (GED)", or "some college or greater". Smoking status was assessed by asking, "Have you smoked at least 100 cigarettes in your lifetime?" Subjects were categorized as smokers if they responded "Yes". Diet was assessed by asking how participants rated their diet with examples (i.e., healthy = high in vegetables, low in fat; unhealthy = high in fat, fast foods) and exercise was assessed with the question. "During the past month, other than your regular job, did you participate in any regular physical activities or exercises?" Responses were categorized as "Yes" or "No".

### Physiologic and clinical measures

Visceral adipose tissue (VAT) was measured using a gated 16-slice CT scanner (Toshiba Aquilon 16, Tustin CA). Eight axial CT images of the abdomen were obtained from each subject, with the sixth slice centered on the L4L5 spinal interspace. The cross-sectional areas of VAT were quantified on each slice using software-derived algorithms (Analyze, version 6.0; Biomedical Imaging Resource, Rochester, MN). The L4L5 VAT measure was used as our dependent variable which is consistent with previous research. Of the 571 participants, VAT measures were obtained on 513 participants and 58 were missing. The absence of VAT measures was due to participant refusal or not showing for their CT scan appointment (n = 36) or inability to conduct the CT scan due to physiological limitations, such as tachycardia, morbid obesity and metal prostheses (n = 22).

Clinical factors included hypertension, diabetes, and lipid status, and history of a first degree relative with heart disease. Fasting (8 hour) blood was collected for serum chemistries and analyzed using a commercial laboratory. History of a first degree relative with heart disease was categorized as yes or no. Hypertension was considered present if the blood pressure was greater than or equal to 140/90 mm Hg for systolic or diastolic pressure, the subject reported being diagnosed with hypertension, or the subject was taking antihypertensive medications. Diabetes was considered present if the fasting glucose level was greater than or equal to 126 mg/dL, the subject reported being previously diagnosed with diabetes, or the subject was taking any diabetic medication. Hyperlipidemia was considered to be present if the participant had a LDL ≥ 160 mg/dL, the participant reported being previously diagnosed with high cholesterol, or the participant was taking a lipid lowering medication.

### Sense of control

**Figure 2:**
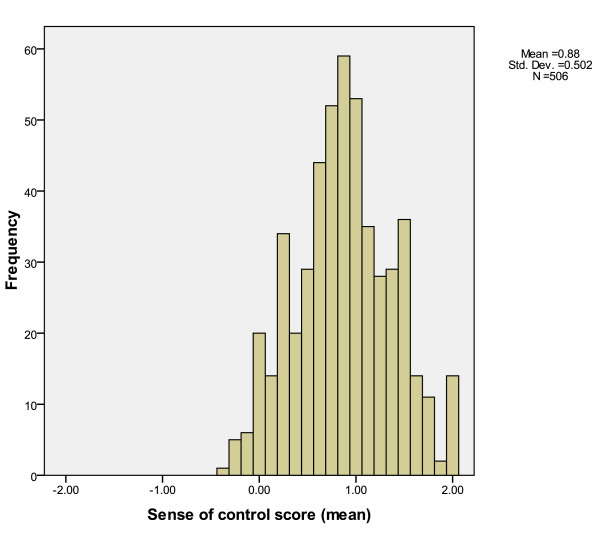
**Sense of control variable distribution histogram**.

The 2 × 2 index of sense of control developed by Mirowsky and Ross was used as the primary independent variable for the current analysis (Cronbach α 0.68) [15]. Sense of control is the extent to which an individual perceives having personal power and direction over outcomes in life. Each participant's overall score was calculated as a mean score from eight questions. Responses to each question included a Likert scale ranging from "Strongly agree" (+2 points) to "Strongly disagree" (-2 points). Higher mean scores indicated a higher sense of control. Sense of control was found to be normally distributed in the study population as depicted in Figure 2.

### Statistical analyses

All statistical analyses were performed using SPSS version 17.0 [21]. Descriptive statistics are provided for all variables. Counts and frequencies are provided for categorical data, and means and standard deviations are provided for continuous variables. Linear regression was performed and unadjusted and adjusted beta-coefficients and standard errors were calculated. Statistical significance was assessed at the alpha = 0.05 level. Chronic conditions and risk factors, such as hypertension, diabetes, and lipid status, are included in the analyses since their relationship with VAT is a complex and dynamic process. While these chronic conditions may be a consequence of unhealthy lifestyles due to poor sense of control, they may also attenuate the development of VAT through the physiologic distribution of excessive glucose and triggering inflammatory response cascades. Therefore, the authors provide adjusted models including and excluding the chronic conditions to assess changes in the overall impact of sense of control on VAT.

**Figure 3:**
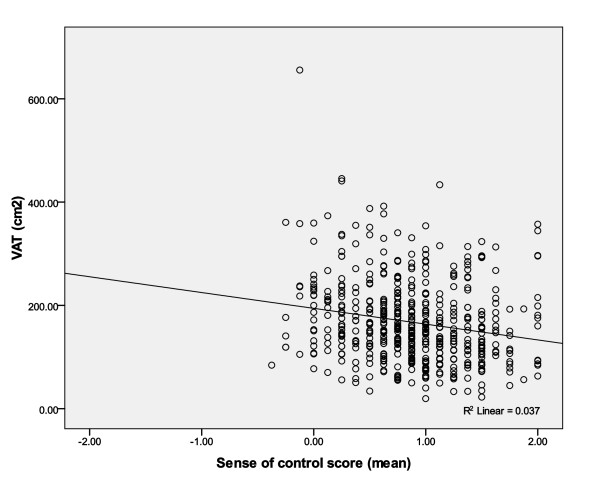
**Scatter-plot for VAT and sense of control with outliers**.

Normality, a required assumption for linear regression analyses, was assessed using stem and leaf plots and skewness and kurtosis statistics for the dependent variable, L4L5 visceral adipose tissue (VAT). After obtaining the natural log of L4L5 VAT (lnVAT), 7 subjects still needed to be excluded from the analyses as they continued to be significant outliers as determined by stem and leaf plots. The final dependent variable used in all analyses was lnVAT. The authors provide a scatter-plot graph (Figure 3) to demonstrate the relationship between VAT and sense of control with the 7 outliers. The adjusted linear regression models also assessed for potential interactions for sense of control*BMI, sense of control*diet, and sense of control*exercise and no interactions were identified. Next, multicollinearity was assessed using Tolerance and Variation Inflation Factor (VIF) with all variables in the final models. No collinear relationships were also identified. The final sample size for all analyses was 506 participants.

## Results

A total of 506 participants were included in the analysis after adjusting for normality. Demographic characteristics are presented in Table 1. For all participants, the median for L4L5 VAT was 351.0 cm^2 ^(IQR = 170.4), and the mean sense of control was 0.8 (sd = 0.5). On average, subjects were 55.2 (sd = 8.1) years of age with a BMI of 31.2 (sd = 6.6). A majority of participants were female (62.5%) and had some college or higher (57.5%). Subjects were non-Hispanic white (27.5%), non-Hispanic African American (32.8%), and Hispanic (38.3%). Of participants, 19.2% had diabetes, 51.8% had hypertension, 52.2% had hyperlipidemia, 49.6% had a first degree relative with a history of coronary heart disease, and 41.7% had smoked 100 cigarettes or more in their lifetime. Approximately, 59% of participants rated their diet healthy, while 70% reported regular physical activity and/or exercise.

**Table 1:** Characteristics of North Texas Healthy Heart study participants -- Fort Worth, Texas, USA 2006-8 (N = 506)

Variable	n*	(%)
Age (years), mean (SD)	55.2	(8.1)

Body mass index (kg/m^2^), mean (SD)	31.2	(6.6)

Sense of control score, mean (SD)	0.8	(0.5)

L4L5 Visceral adipose tissue (VAT, cm^2^), median (IQR)	351.0	(170.4)

Natural log VAT, mean (SD)	5.0	(0.5)

Gender		
Female	316	(62.5)
Male	190	(37.5)

Race/ethnicity		
Non-Hispanic white	139	(27.5)
Non-Hispanic African American	166	(32.8)
Hispanic	194	(38.3)

Education		
Less than High school	113	(22.3)
High school graduate/GED	102	(20.2)
Some college or higher	291	(57.5)

Smoked ≥ 100 cigarettes in one's life		
Yes	211	(41.7)
No	288	(56.9)

Diabetes Mellitus status		
Yes	97	(19.2)
No	387	(76.5)

Hypertension status		
Yes	262	(51.8)
No	124	(24.5)

Hyperlipidemia status		
Yes	264	(52.2)
No	227	(44.9)

First degree relative with history of coronary heart disease		
Yes	251	(49.6)
No	241	(47.6)

Diet rating		
Healthy	299	(59.1)
Unhealthy	204	(40.3)

Regularly exercises in the past month		
No	147	(29.1)
Yes	356	(70.4)

* May not add up to 506 participants due to missing data.

GED, General Equivalency Diploma; IQR, Inter-Quartile Range

Results of simple and multiple linear regressions are presented in Table 2. Sense of control was significantly associated with lnVAT. Without controlling for other factors, an increase in sense of control was associated with a decrease in lnVAT (p < 0.001). After controlling for potential confounders, with and without chronic conditions, the relationship between sense of control and lnVAT remained significant, an increase of sense of control associated with a decrease in lnVAT (p = 0.03), respectively. Furthermore, all adjusted models did not identify any significant effect modifiers including sense of control*BMI, sense of control*diet, and sense of control*exercise. Risk factors positively associated with lnVAT in crude analysis were increasing age (p = 0.008), increasing BMI (p < 0.001), being male (p = 0.001), having less than a high school degree (p < 0.001), having diabetes (p < 0.001), having hypertension (p = 0.001), healthy diet (p < 0.001) and exercise (p = 0.03). Non-Hispanic African Americans had a lower lnVAT than non-Hispanic whites (p < 0.001) in the crude model. In the both adjusted analyses (i.e., with and without chronic conditions in the models), a significant association remained for age (p < 0.001), BMI (p < 0.001), being male (p < 0.001), being non-Hispanic African American (p < 0.001), and diet (p = 0.01 and p = 0.02, respectively).

**Table 2:** Simple and multiple linear regression models predicting natural log of L4L5 visceral adipose tissue (lnVAT) -- Fort Worth, Texas, USA 2006-8 (N = 506)

	Simple linear regression			Multiple linear regression with chronic conditions			Multiple linear regression without chronic conditions		
**Variable**	**β**	**SE**	**p**	**β**	**SE**	**p**	**β**	**SE**	**p**

Sense of control	-0.171	0.040	<.001	-0.077	0.037	0.04	-0.096	0.033	0.004

Age	0.007	0.003	0.008	0.011	0.002	<.001	0.008	0.002	<.001

Body mass index	0.039	0.003	<.001	0.042	0.003	<.001	0.041	0.002	<.001

Gender									
Female	...	...	...	...	...	...	...	...	...
Male	0.140	0.042	0.001	0.163	0.036	<.001	0.188	0.032	<.001

Race/ethnicity									
Non-Hispanic white	...	...	...	...	...	...	...	...	...
Non-Hispanic African American	-0.252	0.050	<.001	-0.358	0.046	<.001	-0.319	0.040	<.001
Hispanic	0.050	0.049	0.31	-0.025	0.049	0.61	0.008	0.043	0.86

Education									
Some college or higher	...	...	...	...	...	...	...	...	...
Less than High school	0.211	0.050	<.001	0.028	0.052	0.60	0.033	0.047	0.48
High school graduate/GED	0.020	0.052	0.69	-0.009	0.045	0.85	-0.015	0.040	0.71

Smoked ≥ 100 cigarettes in one's life									
No	...	...	...	...	...	...	...	...	...
Yes	0.035	0.041	0.39	0.018	0.035	0.61	0.019	0.031	0.54

Diabetes Mellitus status									
No	...	...	...	...	...	...			
Yes	0.244	0.050	<.001	0.051	0.043	0.23			

Hypertension status									
No	...	...	...	...	...	...			
Yes	0.161	0.049	0.001	0.038	0.039	0.33			

Hyperlipidemia status									
No	...	...	...	...	...	...			
Yes	0.075	0.041	0.07	-0.016	0.036	0.65			

First degree relative with history of coronary heart disease									
No	...	...	...	...	...	...	...	...	...
Yes	0.046	0.041	0.27	-0.065	0.035	0.06	-0.047	0.031	0.13

Diet rating									
Healthy	...	...	...	...	...	...	...	...	...
Unhealthy	0.226	0.040	<.001	0.093	0.037	0.01	0.073	0.032	0.02

Regularly exercises in the past month									
No	...	...	...	...	...	...	...	...	...
Yes	-0.099	0.045	0.03	-0.030	0.038	0.44	-0.028	0.034	0.41

β, Beta coefficient; SE, Standard error; p, p-value; ...Reference group; GED, General Equivalency Diploma

## Discussion

Our results revealed that despite controlling for covariates, including traditional cardiovascular risk factors and BMI, sense of control was inversely associated with VAT. Our previous research has found that BMI does not necessarily correlate with VAT when stratified by race/ethnicity [22]. This difference is particularly important since VAT is more metabolically active than other forms of adipose tissue and contributes to metabolic abnormalities associated with weight gain [1-10]. Nonetheless, as expected, BMI was positively associated with VAT measures in our study. Subcutaneous adipose tissue (SAT) was not assessed in the present study for its known direct correlation to BMI and waist conference and being less metabolically active, which is outside the scope of the present study. BMI was assessed as a confounder in our study as it may also be a surrogate measure of unhealthy behaviors. Conversely, it may also function as a mediator in the pathway leading from low sense of control to poor lifestyle and elevated VAT. However, analyses did not find BMI to function as a mediating factor. Another consideration to mention is whether chronic conditions, such as hypertension or diabetes, truly functions as confounders in the present study or rather as final outcomes in the pathway. We chose to assess these chronic conditions as confounders due to the complex and dynamic relationship between VAT and chronic conditions. Chronic disease states, such as elevated sugar, can lead to excessive metabolic burden attenuating the development of VAT or triggering metabolic or inflammatory pathways leading to physiologic dysfunction such as elevated blood pressures and downstream adverse health outcomes. Nonetheless, we presented results with and without chronic conditions in the adjusted regression models and determined it did not alter the association between sense of control and VAT. Our results lend to the importance that one's sense of control or mastery has health consequences that are objectively measured. An increase level of understanding of the pathways by which this occurs continue to be an important area of further research, especially studies that identify factors that are amenable to intervention and improved health outcomes.

Our results align well with other published research in that sense of control is associated with health behaviors and conditions that are normally associated with adiposity [23-25]. High sense of control has been found to be protective in the development of diabetes mellitus among adults [23], while lower levels have been associated with a blunted ability to deal with life stressors possibly leading to poor health behavior decisions [26]. In fact, the relationship between sense of control and VAT is most likely a result of having difficulty in changing poor health behaviors or leading one to make unhealthy choices, which in turn harbors the potential to perpetuate the observation of disparate health outcomes. Low sense of control or mastery has previously been shown to be associated with unhealthy food choices [27], low levels of physical performance [28], and smoking [29]. The multi-factorial attributes of economics, environmental factors, internalizing factors, and social pressures all perpetuate unhealthy behaviors and poorer health. The key to sustainable improved health is not only interventions that impact at the individual level, but also numerous changes associated with the social determinants of health, for example safer communities, policy changes, and financial stability.

There are several limitations to the study that must be considered. While sense of control is related to locus of control, self mastery, and self efficacy, it must be realized that these are related but distinct constructs. Also, the sense of control measure used in this study assessed general control of life choices and was not specific to health. Since the study was conducted in North Texas, USA, generalizability to other locations remains undetermined. In addition, the crude measures for diet and physical activity may not capture the full assessment of each variable. However, the significant findings demonstrated in the simple regression models provide expected associations between these variable and VAT, hence, reassuring the final conclusions of our study. Finally, the cross-sectional nature of the data precludes any causal assumptions.

## Conclusions

Sense of control has been known to be adaptive allowing for focused interventions to function as plausible solutions to change risky and unhealthy behaviors that lead to disease and death. However, the next stages of research need to focus on establishing causality between sense of control and health outcomes. Once causality is established, further interventional research can be performed to assess whether impacting a person's sense of control can lead to improved health behaviors and related outcomes.

## Abbreviations

VAT: visceral adipose tissue; NTHH: North Texas Healthy Heart; NorTex: North Texas Primary Care Practice-Based Research Network; mmHg: millimeters of mercury; kg/m^2^: kilograms per meter squared; lb: pounds; CT: computed tomography; GED: General Educational Development; lnVAT: natural log visceral adipose tissue; VIF: Variation inflation factor; cm^2^: centimeter squared; BMI: body mass index' SE: standard error; SD: standard deviation; β: beta; p: p-value.

## Competing interests

The authors declare that they have no competing interests.

## Authors' contributions

RC conceived of the study, design and analyzed the data, and was the primary writer of the manuscript. KGF and SAH assisted in methodology of the study, assisted in data analyses, edited the manuscript. JC oversaw all labs analyses and methodology and edited the manuscript. All authors read and approved the final manuscript.
